# Targeting metabolic reprogramming in chronic lymphocytic leukemia

**DOI:** 10.1186/s40164-022-00292-z

**Published:** 2022-06-27

**Authors:** Yu Nie, Xiaoya Yun, Ya Zhang, Xin Wang

**Affiliations:** 1 Department of Hematology, Shandong Provincial Hospital, Shandong University, No. 324, Jingwu Road, Jinan, 250021 Shandong China; 2 Department of Hematology, Shandong Provincial Hospital Affiliated to Shandong First Medical University, No. 324, Jingwu Road, Jinan, 250021 Shandong China; 3 Shandong Provincial Engineering Research Center of Lymphoma, Jinan, 250021 Shandong China; 4 Branch of National Clinical Research Center for Hematologic Diseases, Jinan, 250021 Shandong China; 5 National Clinical Research Center for Hematologic Diseases, The First Affiliated Hospital of Soochow University, Suzhou, 251006 China

**Keywords:** Chronic lymphocytic leukemia, Metabolism reprogramming, Lipid metabolism, Targeted therapy

## Abstract

Metabolic reprogramming, fundamentally pivotal in carcinogenesis and progression of cancer, is considered as a promising therapeutic target against tumors. In chronic lymphocytic leukemia (CLL) cells, metabolic abnormalities mediate alternations in proliferation and survival compared with normal B cells. However, the role of metabolic reprogramming is still under investigation in CLL. In this review, the critical metabolic processes of CLL were summarized, particularly glycolysis, lipid metabolism and oxidative phosphorylation. The effects of T cells and stromal cells in the microenvironment on metabolism of CLL were also elucidated. Besides, the metabolic alternation is regulated by some oncogenes and tumor suppressor regulators, especially TP53, MYC and ATM. Thus, the agents targeting metabolic enzymes or signal pathways may impede the progression of CLL. Both the inhibitor of 3-hydroxy-3-methylglutaryl coenzyme A reductase (HMGCR) statins and the lipoprotein lipase inhibitor orlistat induce the apoptosis of CLL cells. In addition, a series of oxidative phosphorylation inhibitors play important roles in decreasing the proliferation of CLL cells. We epitomized recent advancements in metabolic reprogramming in CLL and discussed their clinical potentiality for innovative therapy options. Metabolic reprogramming plays a vital role in the initiation and progression of CLL. Therapeutic approaches targeting metabolism have their advantages in improving the survival of CLL patients. This review may shed novel light on the metabolism of CLL, leading to the development of targeted agents based on the reshaping metabolism of CLL cells.

## Introduction

Chronic lymphocytic leukemia (CLL) is characterized by malignant proliferation of mature monoclonal B lymphocytes in the blood, bone marrow and lymphoid tissues, with heterogeneous outcomes [1]. It is one of the most frequent types of leukemia in adults, which is caused by a complex balance between unrestrained cell proliferation and apoptotic death [2]. Although small targeted agents, for instance, Bruton’s tyrosine kinase inhibitors (BTKi), phosphatidylinositol 3-kinase inhibitors (PI3Ki) and BH3-only mimetics, improve the prognosis of CLL patients, it remains incurable and novel therapeutic options are urgently needed [3–8].

Rewiring of tumor cell metabolism, affected by various tumorigenic alterations, acts as a pivotal avenue to satisfy their needs of survival, malignant proliferation and division [9, 10]. They utilize mass of nutrients and augment biomass synthesis for satisfying energy demands [11, 12]. Metabolic reprogramming of CLL, changing with progression, includes the alterations of glucose metabolism, lipid metabolism and oxidative phosphorylation (OXPHOS) [13, 14]. Of note, recent data have suggested that metabolic reprogramming of CLL can be affected by extensive metabolic interactions with other nonmalignant cells in the microenvironment [15, 16]. Besides, CLL metabolic reprogramming is governed by the expression of aberrant oncogenes and tumor suppressor genes, including TP53, ATM and MYC [17].

To identify therapeutic opportunities specifically targeting metabolism, understanding the role of reprogrammed metabolism in CLL is essential [18, 19]. Herein we recapitulate evidence gathered in recent years regarding metabolic reprogramming in CLL cells, and discuss promising therapeutic strategies in metabolism.

## Intracelluar metabolism

**Fig. 1:**
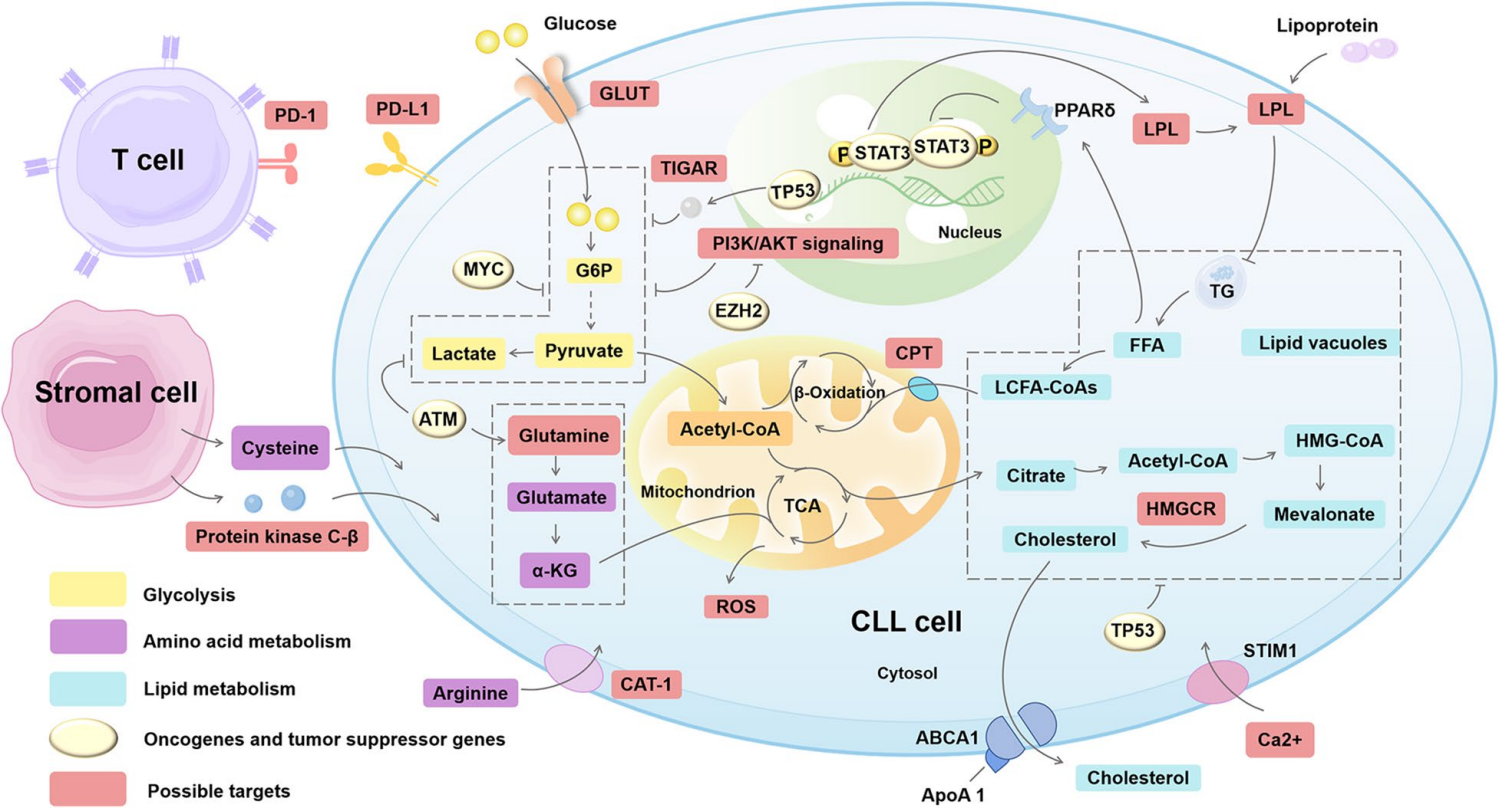
Metabolic reprogramming in chronic lymphocytic leukemia (CLL). In CLL cells, aerobic glycolysis, lipid synthesis, reductive carboxylation, beta-oxidation of fatty acids, and the consumption of glutamine are upregulated. These changes benefit CLL cells as they satisfy their demands of proliferation. *CLL* chronic lymphocytic leukemia, *GLUT* glucose transporter, *G6P* glucose 6-phosphate, *TIGAR* TP53-induced glycolysis and apoptosis regulator, *TCA* tricarboxylic acid, *TG* triglyceride, *LPL* lipoprotein lipase, *LCFA-CoAs* long-chain fatty acyl coenzyme A, *HMG-CoA* 3-hydroxy-3-methylglutaryl coenzyme A, *HMGCR* 3-hydroxy-3-methylglutaryl coenzyme A reductase, *FFA* free fatty acid, *ApoA* apolipoprotein A, *CPT* carnitine palmitoyl transferases, *α-KG* α-ketoglutarate, *CAT-1* cationic amino acid transporter-1, *STIM1* stromal interaction molecule 1, *ROS* reactive oxygen species

Abundant nutrients, including carbohydrates, fats and proteins, represent important drivers of metabolism [20]. Metabolic heterogeneities, induced by internal or external processes to cancer cells, have been found in human cancers, even in distinct regions of the same tumor [18]. Thus, we reviewed the metabolic reprogramming of CLL (shown in Fig. 1).

### Glycolysis

**Fig. 2:**
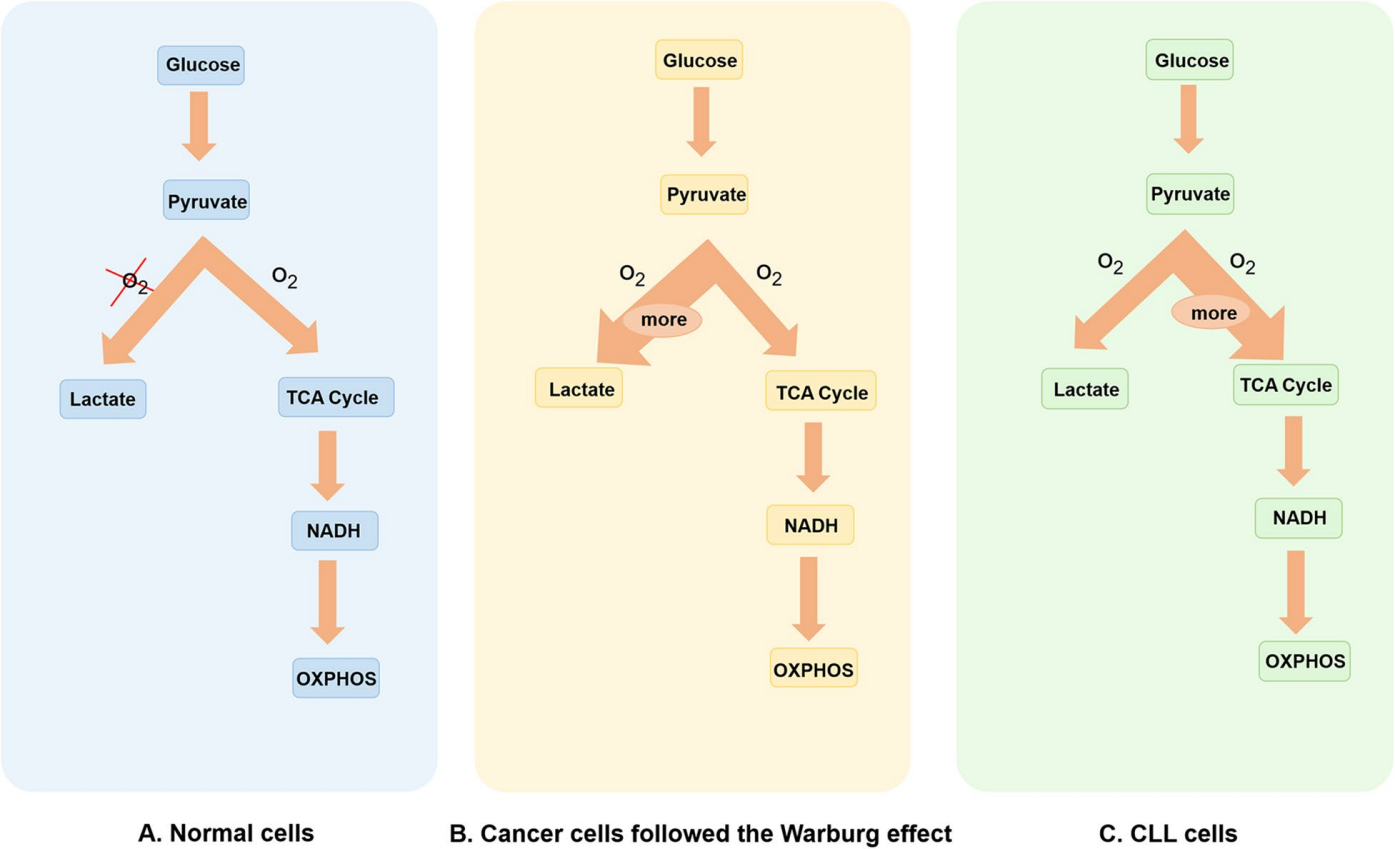
Aerobic glycolysis in CLL cells. **A** In normal cells, glucose is converted to pyruvate, which feeds the tricarboxylic acid (TCA) cycle for energy production under normoxia; **B** Pyruvate predominantly produces energy by lactic acid fermentation, even in the presence of oxygen (aerobic glycolysis) in cancer cells. The flux of pyruvate entering TCA cycle is decreased. **C** CLL cells do not follow the Warburg effect. They are not primarily dependent on glycolysis to produce energy, but increasing mitochondrial oxidative phosphorylation (OXPHOS). *TCA* tricarboxylic acid, *OXPHOS* oxidative phosphorylation

Tumor cells potentiate proliferation by enhancing cellular glucose metabolism [21, 22]. The metabolic intermediates of glycolysis provide cellular energy and play a pivotal role in various macromolecular biosynthesis [19]. Aerobic glycolysis is the principal glucose metabolic pathway in CLL cells. Different from normal cells depending on anaerobic glycolysis in the context of oxygen absence, cancer cells following the Warburg effect display a high proportion of glycolysis even in the presence of oxygen (aerobic glycolysis) [10, 23, 24]. Unlike other rapidly proliferating neoplasms, the energy supply of CLL depends on OXPHOS more than glycolysis (shown in Fig. 2). They increasingly rely on aerobic glycolysis for energy production only under a suitable stimulation, which is activated by the Notch-c-Myc axis partly [25–27]. Recent studies have shown that the primary function of aerobic glycolysis in CLL cells is to maintain high levels of glycolytic intermediates to sustain intracellular anabolic reactions. They participate in many biosynthesis processes, such as the pentose phosphate pathway (PPP) to generate NADPH, ribose-6-phosphate, amino acid, lipids and other cellular sources of energy [23, 28].

Besides, overexpression of glucose transporters (GLUT) facilitates glucose consumption in cancer cells [29, 30]. In glucose metabolism, p53 reduces glucose uptake by repressing the transcription of GLUT-1 and GLUT-4. And p53 suppresses glycolysis by negatively regulating PI3K/AKT signaling [31–33]. In addition, TP53-induced glycolysis and apoptosis regulator (TIGAR) suppresses glycolysis and subsequently prevents intracellular apoptosis reactive associated with oxygen species (ROS). Independently with wild-type p53, the overexpression of TIGAR is correlated with the reduction of spontaneous apoptosis in CLL cells and associated with poor clinical outcomes [34]. Potential therapeutic targets involved in the high level of glucose metabolism need to be further explored.

### Lipid metabolism

Previous studies showed that fatty acid biosynthesis was significantly increased in cancer cells [35, 36]. CLL cells are like adipose cells and muscle cells [37], which can utilize more free fatty acids (FFA) to produce energy compared with normal B-cells or leukemia cells. Lipoprotein lipase (LPL), catalyzing the hydrolysis of triglyceride into FFA, was found aberrant expression in CLL cells. LPL is not expressed in normal lymphocytes, but its expression is significantly increased in IGHV unmutated subset CLL cells [38, 39]. LPL expression and proliferative phenotype in primary CLL B-cells could be induced in the leukemic clone, which promotes malignant B-cell growth [40]. Increased LPL, induced by the activation of signal transducer and activator of transcription-3 (STAT3) or downregulation of microRNA-125, mediates lipoprotein uptake and FFA utilization in CLL cells [41, 42]. LPL could contribute to cancer cell spreading, migration and be involved in CLL progression. The LPL inhibitor orlistat inhibits LPL induced by stimulation of B-cell receptor (BCR) in CLL cells. Treating primary CLL cells with orlistat results in the apoptosis of CLL cells, while no apoptosis is induced in the control group [43].

Lipidomics reveals that CLL cells have aberrant phospholipid levels. CLL membrane may have lower fluidity, resulting in chemotherapy resistance [44]. In addition, carnitine palmitoyl transferases (CPT) support the cell metabolism by transporting FFA into mitochondrial (Mt). CPT1 and CPT2 are upregulated in CLL cells. Perhexiline could inhibit CPT to reduce the cardiolipin, resulting in the damage of Mt membranes and clearing off leukemia cells [45]. Regulation of lipids is a potential therapeutic to improve treatment effects.

Mevalonate (MVA) metabolism not only produces cholesterol, but also contributes to cancer progression, including in the control of cell replication in CLL cells [46]. The statins inhibit 3-hydroxy-3-methylglutaryl-CoA reductase (HMGCR) in the MVA pathway. SREBP2 is the main transcription factor for MVA pathway-associated genes [47]. Tumor suppressor p53 leads to upregulation of the cholesterol-efflux transporter ABCA1, sequentially restricting SREBP2 and repressing the MVA pathway [48]. These results unveil that the MVA pathway has a key role in CLL. Statins are widely used for the prevention and management of cardiovascular disease [49]. In vitro, simvastatin decreases the survival of proliferation and induces apoptosis of CLL cells specifically [41, 50, 51]. It also can enhance the antitumor effect of venetoclax and ibrutinib [50]. Low-potency lipophilic statin could reduce the risk of CLL patients [49]. But statins negatively interfere with rituximab and other anti-CD20 antibodies. The inhibitors of squalene synthase and oxidosqualene cyclase inhibiting cholesterol biosynthesis regulate CD20 expression positively and enhance CLL chemoimmuno-sensitivity and apoptosis [52]. Preclinical studies have indicated that blocking cholesterol synthesis and trafficking blockade could hinder tumor formation and growth [53].

In CLL cells, low-density lipoprotein (LDL) can amplify signaling pathways, particularly cytokine-signaling [54]. Reducing LDL levels inhibits signaling and limits the proliferation of CLL cells. Besides, researchers have reported that the level of high-density lipoprotein (HDL) was decreased in patients with CLL [55]. Apolipoprotein A (ApoA) is one of the main components of HDL. A low level of ApoA is related to the advanced stage of CLL patients [56]. ApoA1 mimetics could inhibit the proliferation of CLL cells. ApoA5 has been found to play a significant function in triglyceride metabolism by stimulating LPL activity [57]. The role of ApoA in CLL requires additional investigation.

### Amino acid metabolism

Amino acids are widely rewired to obtain the necessary energy to satisfy the increased need in cancer cells [58–61]. The level of isoleucine is decreased, while pyruvate and glutamate are increased in CLL patients [62, 63]. Among amino acids in vivo, glutamine (Gln) metabolism is important for cancer cell survival, which is the central position in carbon and nitrogen metabolism in tumor cells [64]. Previous research indicated the consumption of Gln was increased in CLL [65]. Elevated ammonia uptake and Gln synthetase expression are found in del11q CLL lymphocytes, favoring de novo Gln synthesis [66]. The expression of glutamate dehydrogenase (GDH) is decreased in del11q CLL cells, which benefits transaminase reactions using α-ketoglutarate for glutamate synthesis and reduces oxidative deamination of glutamate. Recent studies have suggested that the activity of membrane mechanistic target of rapamycin complex 1 (mTORC1) can be stimulated by extracellular Gln, which facilitates the transport of mTORC1-activating amino acids across the plasma. Apart from regulating mTOR, L-glutamate regulates translation to coordinate cell growth and proliferation [67]. However, ammonia, as a by-product of glutaminolysis, stimulates autophagy in a mTORC1-independent fashion [68]. Depending on these alterations, based on metabolism therapeutic options could be developed. Many novel mTOR inhibitors are being explored in clinical trials [69]. Affecting glutamine metabolism is one of the mechanisms of ibrutinib. Glutaminase inhibitor L-asparaginase catalyzes the conversion of Gln to glutamate in CLL cells, especially in those with del11q [70]. To conclude, the specific Gln metabolism characteristics in CLL are worth further study.

Besides Gln, arginine also plays an important role in CLL. Certain tumors have been verified to lose the ability to synthesize arginine independently, which is promising to be a therapeutic target [71, 72]. Argininosuccinate synthase (ASS) is not expressed in primary CLL cells, preventing arginine synthesis. As the only arginine importer expressed in CLL cells, cationic amino acid transporter-1 (CAT-1) is a novel target for CLL therapy [73]. The abnormal activation of amino acids maintains the metabolic balance in CLL.

### Ion metabolism

One of the ion metabolic peculiarities of CLL is Ca^2+^ dysregulation [74]. The level of basal Ca^2+^ signaling is not uniformly increased in CLL cells. Compared with normal B cells, basal Ca^2+^ signaling in CLL cells is higher, especially in IGHV mutated CLL (M-CLL) [75]. Recent studies have shown a novel Ca^2+^ entry pathway, named constitutive Ca^2+^ entry (CE), which is constitutive and BCR-independent, is controlled by stromal interaction molecule 1 (STIM1) located at the plasma membrane [74]. Besides, the surface protein CD38 can enhance intracellular Ca^2+^ levels to promote RasGRP2/Rap1-mediated CLL cell adhesion and migration [76, 77]. Pathways associated with calcium ions, such as Rap1 signaling, may lead to potential therapeutic strategies targeting CLL treatment. Calcium concentration variation is directly through the BCR and chemokine receptors, or indirectly through co-stimulatory molecules. Transmit information depending on calcium signals is crucial to B-cell ontogeny, including specific signaling pathways essential for B cells development and activation [78, 79].

In addition, iron plays dual roles in the CLL cells. On the one hand, iron is a critical cofactor that is required for DNA synthesis [80]. The transferrin receptor (TfR) contributes to iron import and a higher TfR concentration has been found in CLL directly reflecting the large tumor burden [81]. Leukemia cells harbored TP53 mutation need a mass of iron to support rapid proliferation. P53 plays a role in iron homeostasis and mitochondrial iron homeostasis by modulating iron regulators [82]. Cells may go into canceration or demise when the homeostasis is disrupted [83]. Ferroptosis, a crucial component of p53-mediated tumor suppression, is an iron-dependent form of regulated cell death caused by unrestricted lipid peroxidation and subsequent plasma membrane rupture [84]. The activity of the selenoperoxidase Glutathione Peroxidase 4 (GPX4) is the cornerstone of the antiperoxidant defence [85]. The expression of GPX4 in ferroptosis depends on the presence of glutathione (GSH) [86]. Eprenetapopt, which could reactivate mutant forms of p53 and induce ferroptosis by GSH depletion, is currently being tested in the clinical trials involving patients with acute myeloid leukemia (NCT03931291) [87]. Cysteine availability is the main limiting factor in the synthesis of GSH. The depletion of extracellular cysteine leads to cell death in CLL. Cyst(e)inase, an engineered human enzyme, effectively degrades cysteine and induces ferroptosis in pancreatic ductal adenocarcinoma [86]. The strategy regulating extracellular cysteine levels opens up new therapeutic options for CLL patients with TP53 mutation. Nevertheless, the definitive mechanism that p53 modulates iron metabolism in CLL is unclear. Targeting altered iron metabolic pathway is specific to CLL patients with TP53 mutation.

## Mitochondrial metabolism

Mt plays an important role in cellular metabolism, ATP synthesis, oxidative metabolism and the regulation of apoptosis [88, 89]. Compared with solid tumor cells, aerobic glycolysis is not increased in CLL cells, while the level of mitochondrial OXPHOS is elevated. Mt biogenesis, such as Mt mass, ATP production, Mt DNA, oxygen consumption and the production of ROS, has increased in CLL cells. The accumulation of ROS may contribute to the metabolic state of oxidative stress in CLL patients [90–92].

OXPHOS is upregulated in leukemias, including CLL. OXPHOS inhibitors could be used to improve treatment outcomes [93]. Recent studies have identified that PI3K/AKT signaling could be limited by the suppression of the expression and activity of the inhibitory phosphatase SH2-containing-inositol-5′-phosphatase-1 (SHIP1) in CLL cells. Increased Mt respiration and excessive accumulation of ROS lead to CLL cells death [94]. Besides, mtDNA mutations elevate the level of nitric oxide (NO), which can significantly influence Mt biogenesis. By inhibiting the expression of NO synthases (NOS), which can be induced by ROS stress, the NO-mediated Mt biogenesis in CLL cells can be changed [95]. Previous study verified the ability of 22 NOS inhibitors to induce CLL cell apoptosis, including L-NAME [96]. In addition, the Ser727-phosphorylated STAT3 molecule (pSTAT3Ser727) in Mt overactivity can enhance the antioxidant defense ability of CLL B cells that promotes their survival [97, 98].

Hence, recent studies have shown that combinations with mitochondrial targeting agents could be a promising cancer therapy. Of note, some unanticipated agents regulating metabolism also play roles in CLL. The OXPHOS inhibitor metformin (NCT01750567) alone or in combination with the GLUT4 inhibitor ritonavir (NCT02948283) was involved in the current clinical trials [70]. Metformin inhibits the OXPHOS of mitochondrial in CLL cells, while ritonavir induces CLL cells apoptosis [99]. The OXPHOS inhibitor IACS-010759 inhibits OXPHOS and diminishes intracellular ribonucleotide pools [100]. Berberine (BRB), used for metabolic disorders, induces impairment of OXPHOS and the associated increment of oxidative damage, with consequent inhibition of CLL cell activation and eventual cell death [101]. Besides, PK11195, the benzodiazepine derivate, blocks OXPHOS and induces apoptosis in CLL [102].

## Role of the microenvironment in CLL metabolism

The specific microenvironment can promote the survival of CLL cells. In most human cancers, non-malignant cells in the microenvironment limit oxygen and nutrient transport to the cancer cells [36, 103]. Lipid metabolism in microenvironment plays the paradoxical role in anti-tumor and pro-tumor immune responses [104]. Therefore, cancer cells transfer their metabolic ability and adapt their microenvironment to support cancer cell growth and satisfy their biomass demands, even involving in cancer metastasis [105, 106].

The microenvironment activates and protects CLL cells through several mechanisms [107]. CLL cells, primarily existing in peripheral blood and tissues and proliferate mainly in lymph nodes, interact directly with T cells, monocyte-derived cells (MDC) and stromal cells in proliferation centers. The signals, such as adhesion molecules, cell surface ligands, chemokines, cytokines and their respective receptors, mediate the interactions between CLL cells and the microenvironment. These signals promote an immunotolerant milieu in the CLL lymph node [108]. Thus, the microenvironment affects the metabolism of CLL cells significantly.

In CLL patients, T cells can activate mitochondrial metabolism, enhance chemo-resistance and promote cell proliferation. Follicular helper T cells (T_FH_) populations expand abnormally and produce a mass of cytokines and costimulatory molecules to help CLL cells proliferate [109, 110]. Besides, CLL cells express high levels of PD-L1 while T cells express PD-1 [111]. Blocking PD-1/PD-L1 might restore the glycolysis, phagocytosis and BTK signaling of monocytes/macrophages in CLL [112]. Direct contact with CLL cells induces T-cell dysfunction [113], such as inducing Rho GTPase signaling defects in T cells to evade immune recognition [114].

Stromal cells prolong the survival time of CLL cells by generating stromal cell-derived factor-1 (SDF-1) [115]. The survival signals delivered by stromal cells activate different pathways. Mesenchymal stromal cells (MSCs) in CLL patients signifcantly promote CLL cell proliferation compared with the control group [116]. In addition, MSC-derived extracellular vesicles (EVs) increase CLL cell migration and give CLL cell survival advantages [117]. Bone marrow stromal cells take in cystine and transform it into cysteine, which is transported to CLL cells for glutathione synthesis and enhances survival and drug resistance of CLL cells [118]. The selective inhibitor of the lipid kinase PI3Kδ idelalisib (NCT01539512) promotes apoptosis in primary CLL cells, and disrupts interactions between CLL cells and endothelial cells/bone marrow stromal cells [119, 120]. Bone marrow stromal cells induce the production of protein kinase C-β to promote CLL progression [121]. Protein kinase C-β lessens mitochondrial stress and facilitates glucose absorption. Disrupting this bidirectional communication between CLL cells and stromal cells can study novel treatment strategies.

## Gene expression of metabolic reprogramming in CLL

Aberrant expression of oncogenes and tumor suppressors facilitates the metabolic reprogramming of cancer cells to enable increased nutrient acquisition and biosynthesis [15]. However, how gene expression affects metabolism of CLL cells is less understood. Therefore, we summarized the impact of gene expression on metabolic reprogramming of CLL cells (shown in Table 1).

**Table 1:** Oncogenes and tumor suppressor genes participate in the metabolic reprogramming of chronic lymphocytic leukemia (CLL)

Gene	Effects on metabolic pathways	Relevance to CLL
TP53	Glucose metabolism Lipid metabolism OXPHOS Iron metabolism	TP53 plays key roles in cell cycle arrest, apoptosis, DNA repair and autophagy. The TP53 mutation/deletion is a poor prognostic biomarker in CLL, and tailors the therapy of CLL patients
ATM	Glutamine metabolism Glucose metabolism	ATM mutations predict for shorter time to first treatment irrespective of the IGHV mutation status
MYC	Glutamine metabolism Glucose metabolism	Mutations in MYC have been linked to Richter syndrome. BCR engagement enhances MYC expression in a BTK dependent manner as it is abrogated by ibrutinib
SI	Carbohydrate metabolism	SI participants in metabolic reprogramming in CLL cells
AKT	Glucose metabolism	Active AKT signaling triggers CLL toward Richter transformation via overactivation of Notch1
EZH2	Glutamine metabolism Lipid metabolism Amino acid metabolism	EZH2 upregulates the PI3K/AKT pathway through IGF1R and MYC in aggressive CLL

The human tumor suppressor gene TP53, located on the short arm of chromosome 17, plays key roles in cell cycle arrest, apoptosis, DNA repair, autophagy and metabolism regulation in CLL [122, 123]. TP53 mutation and/or deletion of chromosome 17 is a poor prognostic biomarker and tailors the therapy in CLL patients [124–126]. Importantly, the protein p53, targeting various metabolism pathways, enables cells to maintain metabolic homeostasis and adapt to stress [127]. Researchers have indicated that mutant TP53 could conserve its tumor suppressor activity by decreasing reactive oxygen production and regulating energy metabolism [128]. In lipid metabolism, p53 inhibits fatty acid synthesis and even enhances fatty acid oxidation in cells [129]. Besides, p53 takes effect on OXPHOS, mitochondrial metabolism, serine metabolism, nucleotide metabolism and iron metabolism [129, 130]. In lymphomas, cellular metabolism and metabolic stress influence the activity of p53 conversely. Metabolic stress induced by glucose deprivation leads to cell-cycle arrest and apoptosis. The Gln metabolism and glycolysis also affect the transcription activity of the p53 protein [131]. Taken together, TP53 is a critical gene in CLL cellular metabolism and moderated by the metabolic status of the cells.

The MYC oncogene family, including c-MYC, N-MYC and L-MYC, encodes a group of nuclear phosphoproteins that take effect in cell proliferation, apoptosis and progression of cancers [132]. Endogenous and oncogenic MYC appears to share target genes involved in several facets of intermediary metabolism from glycolysis and glutaminolysis to nucleotide and lipid synthesis [133]. It was reported that the metabolic genes would be further amplified to support the bioenergetic needs of the growing cells when MYC is induced. In proliferative tumor cells, MYC increases the expression of glutamine transporters and glutaminase to promote mitochondrial glutamine utilization [134]. Through upregulation of enzymes in the PPP, MYC increases the shunting of glucose to the PPP in lymphocytes [135]. In addition, it regulates lymphocyte serine biosynthesis. Besides, MYC activates the expression of the enzymes ATP citrate lyase (ACLY), acetyl-CoA carboxylase alpha, fatty acid synthase (FASN) and stearoyl-CoA desaturase (SCD), involved in fatty acid synthesis from citrate [136, 137]. However, whether MYC takes similar effects in CLL needs further exploration. The expression of MYC in CLL is increased with disease progression, independent of other prognostic factors [70]. Richter syndrome (RS) is the transformation of CLL into an aggressive lymphoma and has a poor prognosis [138]. MYC aberrations are common in RS and enhanced glucose metabolism is detected in RS [139]. MYC may upregulate the glucose metabolism in CLL cells leading to disease progression.

The human tumor suppressor gene ATM, located on the long arm of chromosome 11, relates to DNA damage, cell cycle progression, p53 dysfunction and metabolism [66, 140]. In CLL, the ATM mutation is a poor independent prognostic biomarker for time to first treatment (TTFT) [141]. The underexpression of ATM is associated with the deletion of chromosome 11 (del11q), while del11q CLL lymphocytes reprogram glutamine metabolism and inhibit glucose metabolism [66]. Besides, increased expression of insulin receptors is found in del11q CLL [142]. Thus, del11q, associated with the low expression of ATM, inhibits glucose and glutamine metabolism pathways.

Besides the tumor suppressor genes and oncogenes above, other genes also take metabolic effects in CLL. Sucrase-isomaltase (SI) is a carbohydrate metabolism enzyme and SI gene mutations involve in the metabolism and development of cancer [143, 144]. In CLL, SI mutations result in metabolic reprogramming of glucose, heterocycle and cofactor metabolism. In this respect, SI could be an overlooked cancer gene. The oncogene AKT stimulates glucose metabolism and lactate production without increasing oxygen consumption in glioblastoma and hematopoietic cancer cell [145]. Active AKT signaling triggers CLL toward RS [146, 147]. While enhanced glucose metabolism is found in RS, the role of AKT plays in the glucose metabolic pathway of CLL may need further study. Due to the difference in cellular context, EZH2 acts as either an oncogene or a tumor suppressor gene [148, 149]. It alters metabolism of cancer cells involving glucose, lipid and amino acid metabolism [150]. In aggressive CLL, EZH2 upregulates the PI3K/AKT pathway through IGF1R and MYC, thus regulating glycolysis, glutaminolysis and mitochondrial biogenesis [151, 152]. Overexpression of EZH2 is associated with a poor prognosis of CLL [153]. Overall, some oncogenes and tumor suppressor genes could regulate metabolic reprogramming in CLL.

In the last decade, the biological basis of CLL pathogenesis studies has greatly expanded our knowledge of the progression of CLL remarkably, revealing a huge number of novel alterations that might drive the evolution of the disease. Therefore, we summarized the reprogrammed pathways or biomarkers related to metabolism in CLL (shown in Tables 2 and 3).

**Table 2:** Reprogrammed signaling pathways associated with metabolism in CLL

Pathways	Mechanism of action
NF-κB signaling pathway	Constitutively activated and interacts with BCR, Toll-like receptors and CD40 in CLL
PI3K/AKT/mTOR pathway	Constitutively activated and plays a pivotal role in the aberrant OXHPHOS and glycolysis and involves in CLL cell survival and migration
Notch-c-Myc signaling pathway	Increases aerobic glycolysis in CLL cells activated by bone marrow stromal cells
BCR signaling pathways	Engages glucose and glycerol metabolism

**Table 3:** Biomarkers in reprogrammed pathways related to metabolism in CLL

Biomarkers	Mechanism of action
BTK	Involves in CLL cell proliferation and adhesion, BCR signaling, chemokine secretion (CCL3, CCL4)
ZAP-70	Overexpressed in CLL cell and enhances BCR signaling
Spleen tyrosine kinase (SYK)	Involves CLL cell survival and migration via BCR and chemokine receptor signaling
Lyn	Overexpressed in CLL cell as a major contributor to antigen-independent BCR signaling
Sprouty2	Significantly decreased in CLL cells from poor-prognosis patients and attenuates BCR and MAPK-ERK signaling in CLL cell
CD5	Promotes the activation of the PI3K/Akt/mTOR and MAPK-ERK pathway
CD40	Regulates amino acid metabolism, TCA and energy production

## Other therapies targeting metabolic pathways in CLL

As mentioned, CLL cells alter the metabolic pathways to satisfy the need for proliferation and survival. In this context, metabolism is a novel target for CLL patients (detailed information is shown in Table 4).

**Table 4:** Possible effects of metabolism-associated agents in CLL

Agents	Primary mechanism	Possible effects in CLL	Identifier
Statins	Competitive inhibitors of HMG-CoA reductase	Induces apoptosis of CLL cells	–
Orlistat	LPL inhibitor	Induces apoptosis of CLL cells	–
Idelalisib	Selective inhibitor of the lipid kinase PI3Kδ	Promotes apoptosis in primary CLL cells Disrupt interactions between CLL with endothelial cells and bone marrow stromal cells	NCT01539512
Ratonavir	GLUT4 inhibitor	Induces apoptosis	NCT02948283
L-asparaginase	Glutaminase inhibition	Catalyzes the conversion of glutamine to glutamate, especially in del11q CLL cells	–
L-NAME	NOS inhibitors	Induces CLL cells apoptosis	–
Berberine	Isoquinoline alkaloid	Inhibits CLL cell activation and eventual cell death	–

Besides the treatments discussed above, the potential agents on the suite of CLL metabolism need more research to prove. For instance, a cardiac glycoside is a therapy for heart failure and arrhythmia. Ipecac alkaloids are used as anti-infective. They both repress hypoxia-inducible factor-1α (HIF-1α) and disturb intracellular redox homeostasis in CLL cells, as well as highly active against protected primary CLL cells [154, 155].

Ibrutinib, the BTK inhibitor, is the first choice for CLL [125]. In addition to targeting BCR signaling, ibrutinib participants in the control of lipid and mitochondrial metabolism [70]. Increased HDL level is found in CLL patients received ibrutinib therapy [156]. Ibrutinib affects Mt metabolism, and curtailing AMPK activity might sensitize ibrutinib-resistant clones to ibrutinib [157]. These results indicate that ibrutinib induces bioenergetic stress responses.

## Metabolic reprogramming in other hematological malignancies

Metabolic heterogeneity is found in acute myeloid leukemia (AML) at different stages. Human leukemia stem cells (LSC) are dependent on OXPHOS for survival regulated by AMPK, as well as mTOR. Amino acid uptake, steady-state levels and catabolism are elevated in the LSC [158, 159]. Ecotropic Virus Integration site 1 protein homolog (EVI1) induces accelerated OXPHOS prior to activation of glycolysis in mixed lineage leukemia-rearrangement AML, with a higher dependency on Gln [160]. On the other hand, fatty acids oxidation (FAO) is a key metabolic pathway fostering the survival of chemoresistant LSC. Inhibiting very-long-chain acyl-CoA dehydrogenase, which supports FAO and OXPHOS in the mitochondrial metabolism in AML, is demonstrated preclinical activity [161]. In contrast to LSC, bulk AML blast cells rely on glycolysis to produce energy, and upon glucose deprivation have decreased viability in culture [59]. High expression of phosphomannonse isomerase (PMI), as a poor prognostic factor of AML, mobilizes mannose to glycolysis under glucose starvation in leukemia [162]. In addition, thioredoxin reductase (TrxR) directly regulates GAPDH leading to a disruption of glycolysis and an increase in flux through the PPP [163]. Combined inhibition of TrxR and PPP leads to leukemia growth inhibition.

Similar to lymphomas and AML, aberrant glycolysis and OXPHOS are the main altered metabolic processes in multiple myeloma (MM). Lactate dehydrogenase isoform A (LDHA), a key enzyme in glycolysis, is highly expressed in MM [164]. In MM, the PPARγ coactivator-1β (PGC1β) promotes the transcription of LDHA, thus modulates glycolysis and mitochondrial function. Otherwise, MM cells with overexpression of PLR-3 have higher aerobic glycolytic rate, OXPHOS and ATP production by promoting glucose uptake and lactate excretion, enhancing the levels of proteins regulating glycolysis and enzymes in the serine/glycine synthesis pathway [165]. Besides, lipid metabolism and microenvironment affect the cell proliferation in MM. In bone marrow, MM cells induce lipolysis of adipocytes. Subsequently, released FFA is taken up by myeloma cells through FFA transporter proteins, leading to growth or lipotoxicity [166].

One of the common metabolic characteristics among some B-cell derived lymphomas is the increased OXPHOS. According to consensus cluster classification, diffuse large B-cell lymphoma (DLBCL) is separated into three clusters with distinct metabolic fringerpringts: OXPHOS-DLBCL, BCR-DLBCL and host response-DLBCL [167]. OXPHOS-DLBCL displays a prominent mitochondrial component with elevated OXPHOS. Quantitative evidence validates marked increased mitochondrial FAO and palmitate is a predominant respiratory fuel in OXPHOS-DLBCL [168]. In contrast, non-OXPHOS-DLBCL is metabolically rewired to aerobic glycolysis. In addition, overexpression of transporters of lactate in DLBCL cells, such as monocarboxylate transporter 1 (MCT1) and TOMM20, promotes the TCA cycle of malignant cells in the process of reverse Warburg effect [169]. Similarly, Hodgkin and Reed Sternber cells with high expression of MCT1 and TOMM20 are of increased mitochondrial metabolism, while tumor-associated macrophages with high expression of MCT4 present elevated glycolysis in the microenvironment [170]. mTOR signaling plays a pivotal role in the aberrant OXHPHOS and glycolysis in B-cell derived lymphoma, including DLBCL, follicular lymphoma (FL), mantle cell lymphoma (MCL) [171, 172]. However, rapalogs, the inhibitor of mTORC1, fails to improve the prognosis of refractory/relapsed DLBCL, FL and MCL in the clinical studies [171]. mTOR signaling pathway is a potential therapeutic target for the B-cell derived lymphomas.

To some extent, the metabolism rewiring of CLL is in accordance with partly B-cell derived lymphomas, in which the survival and proliferation of cells depend on elevated OXPHOS or aerobic glycolysis. Although metabolic alternations of other hematological malignancies involve in lipid metabolism, the overexpression of LPL is not reported in the hematological malignancies except for CLL. Similar to adipocytes, lipid-like vesicles uptaked by LPL are detected in CLL cells [42]. Several proteins that drive steps in glycolysis and FFA biosynthesis are overexpressed, while proteins involved in the citric acid cycle are at low levels in CLL cells. This suggests CLL cells increase both endogenous lipid synthesis and exogenous lipid uptake [44]. Compared to other hematological malignancies, CLL cells are more inclined to rely on lipid metabolic pathways to support their survival.

## Conclusions

Extensive research over the last decade has provided compelling evidence for metabolic reprogramming in CLL. Unlike normal cells which rely on aerobic glucose metabolism, CLL cells depend on the aberrant lipid metabolism and mitochondrial OXPHOS to support survival and growth. Altered metabolism of amino acids and ions contribute to the intracellular signal transduction in CLL cells. In addition, the microenvironment, activating the metabolism through several mechanisms, plays a vital role in the survival of CLL cells. Besides, the process of metabolic rewiring in CLL is driven by some oncogenes and tumor suppressor genes, especially TP53, MYC and ATM. Exploring novel agents with high selectivity and specificity by regulating the metabolic activity provides an opportunity to benefit CLL patients. Furthermore, the synergistic effects of metabolism-regulated drugs with targeted therapy need to be tested comprehensively in the clinic to facilitate development of novel treatment strategies for CLL patients. With improved understanding of CLL metabolic reprogramming, the rise of innovative therapeutic interventions that target metabolic pathways is anticipated.

## Data Availability

Not applicable.

## Abbreviations

CLL: Chronic lymphocytic leukemia
BTKi: Bruton’s tyrosine kinase inhibitor
FFAs: Free fatty acids
GLUT: Glucose transporter
PPP: Pentose phosphate pathway
OXPHOS: Oxidative phosphorylation
LPL: Lipoprotein lipase
STAT3: Signal transducer and activator of transcription-3
HDL: High-density lipoprotein
MVA: Mevalonate
HMGCR: 3-Hydroxy-3-methylglutaryl-CoA reductase
ApoA: Apolipoprotein A
Gln: Glutamine
GDH: Glutamate dehydrogenase
ASS: Argininosuccinate synthase
CAT-1: Cationic amino acid transporter-1
ROS: Reactive oxygen species
STIM1: Stromal interaction molecule 1
Mt: Mitochondrial
CPT: Carnitine palmitoyl transferases
SHIP1: SH2-containing-inositol-5′-phosphatase-1
BRB: Berberine
mTORC1: Membrane mechanistic target of rapamycin complex 1
NO: Nitric oxide
NOS: Nitric oxide synthase
CE: Constitutive Ca^2+^ entry
TfR: Transferrin receptor
GPX4: Glutathione peroxidase 4
GSH: Glutathione
pSTAT3Ser727: Ser727-phosphorylated STAT3 molecule
MDC: Monocyte-derived cells
T_FH_: Follicular helper T cells
ACLY: ATP citrate lyase
FASN: Fatty acid synthase
SCD: Stearoyl-CoA desaturase
SDF-1: Stromal cell-derived factor-1
HIF-1α: Hypoxia-inducible factor-1α
MSCs: Mesenchymal stromal cells
EVs: Extracellular vesicles
TIGAR: TP53-induced glycolysis and apoptosis regulator
TTFT: Time to first treatment
RS: Richter syndrome
SI: Sucrase-isomaltase
SYK: Spleen tyrosine kinase
DLBCL: Diffuse large B-cell lymphoma
FAO: Fatty acids oxidation
FL: Follicular lymphoma
MCL: Mantle cell lymphoma
AML: Acute myeloid leukemia
LSC: Leukemia stem cell
EVI1: Ecotropic Virus Integration site 1 protein homolog
PMI: Phosphomannonse isomerase
TrxR: Thioredoxin reductase
MM: Multiple myeloma
MCT: Monocarboxylate transporter
LDHA: Lactate dehydrogenase isoform A
PGC1β: PPARγ coactivator-1β
HMG-CoA: 3-Hydroxy-3-methylglutaryl coenzyme A
BCR: B-cell receptor
